# From a case-control survey to a diagnostic viral gastroenteritis panel for testing of general practitioners’ patients

**DOI:** 10.1371/journal.pone.0258680

**Published:** 2021-11-03

**Authors:** Lesla E. S. Bruijnesteijn van Coppenraet, Jacky Flipse, Janny A. Wallinga, Marloes Vermeer, Wil A. van der Reijden, Jan F. L. Weel, Adri G. M. van der Zanden, Theo A. Schuurs, Gijs J. H. M. Ruijs

**Affiliations:** 1 Laboratory of Medical Microbiology and Infectious Diseases, Isala, Zwolle, The Netherlands; 2 ZGT Academy, Ziekenhuisgroep Twente, Almelo, The Netherlands; 3 Regional Laboratory for Medical Microbiology and Public Health Kennemerland, Haarlem, The Netherlands; 4 Izore, Center for Infectious Diseases Friesland, Leeuwarden, The Netherlands; 5 Laboratory for Medical Microbiology and Public Health Labmicta, Enschede, The Netherlands; GGD Amsterdam, NETHERLANDS

## Abstract

**Objective:**

To evaluate the pathogenicity of a broad range of 11 possible gastroenteritis viruses, by means of statistical relationships with cases *vs*. controls, or Ct-values, in order to establish the most appropriate diagnostic panel for our general practitioner (GP) patients in the Netherlands (2010–2012).

**Methods:**

Archived stool samples from 1340 cases and 1100 controls were retested using internally controlled multiplex real-time PCRs for putative pathogenic gastroenteritis viruses: adenovirus, astrovirus, bocavirus, enterovirus, norovirus GI and GII, human parechovirus, rotavirus, salivirus, sapovirus, and torovirus.

**Results:**

The prevalence of any virus in symptomatic cases and asymptomatic controls was 16.6% (223/1340) and 10.2% (112/1100), respectively. Prevalence of astrovirus (adjusted odds ratio (aOR) 10.37; 95% confidence interval (CI) 1.34–80.06) and norovirus GII (aOR 3.10; CI 1.62–5.92) was significantly higher in cases versus controls. Rotavirus was encountered only in cases. We did not find torovirus and there was no statistically significant relationship with cases for salivirus (aOR 1,67; (CI) 0.43–6.54)), adenovirus non-group F (aOR 1.20; CI 0.75–1.91), bocavirus (aOR 0.85; CI 0.05–13.64), enterovirus (aOR 0.83; CI 0.50–1.37), human parechovirus (aOR 1.61; CI 0.54–4.77) and sapovirus (aOR 1.15; CI 0.67–1.98). Though adenovirus group F (aOR 6.37; CI 0.80–50.92) and norovirus GI (aOR 2.22, CI: 0.79–6.23) are known enteropathogenic viruses and were more prevalent in cases than in controls, this did not reach significance in this study. The Ct value did not discriminate between carriage and disease in PCR-positive subjects.

**Conclusions:**

In our population, diagnostic gastroenteritis tests should screen for adenovirus group F, astrovirus, noroviruses GI and GII, and rotavirus. Case-control studies as ours are lacking and should also be carried out in populations from other epidemiological backgrounds.

## Introduction

Acute diarrhea is a frequent cause of morbidity in the general population. In The Netherlands approximately 4.5 million people a year experience an episode of gastroenteritis [1]. In 2019, 294,100 Dutch patients out of a population of 17.2 million visited their family physician (GP) because of complaints of diarrhea or gastrointestinal (GI) complaints. Diarrhea may manifest as loose stools or, occasionally, with nausea, vomiting and fever [2]. Often self-limiting, testing is rarely indicated, but should be considered in more serious cases, immunocompromised patients, or epidemical situations [1]. Because the pathogen cannot be deduced from symptoms alone, laboratory diagnostics are essential. Since there were no data of recently identified putative gastroenteritis pathogens in the general population of a high-income-country (HIC) such as The Netherlands, we conducted a case-control study involving bacterial, protozoal and viral pathogens. Following our analyses on the bacterial and protozoal agents we now report on the viruses [3,4].

A broad viral panel was composed for testing by quantitative real time PCR (qRT-PCR), including all putative pathogenic viruses associated with gastroenteritis in humans: adenovirus, astrovirus, bocavirus, enterovirus norovirus GI and GII, parechovirus, rotavirus, salivirus, sapovirus, and torovirus [5–13]. Results were analyzed for statistically significant relationships between the respective virus and the prevalence in cases vs. controls, assuming that a statistically significant relationship of <0,05 is indicative of pathogenicity.

However, a positive test result does not always indicate the cause of the diarrheal complaints. It can also be false-positive, originating from a patient who was previously asymptomatically colonized by a pathogen and became ill for another unrelated reason. Asymptomatic carriership can vary by population. For example, the epidemiological background of a GP population differs from that of a quaternary care environment. Hence, case-control studies are needed for interpreting positive findings in the population in which the assay of interest will be deployed. Moreover, case-control studies can contribute significantly to discussions on the possible pathogenicity of gastrointestinal micro-organisms, using statistically significant correlation with cases, or even controls, as contributing arguments [3,4,14]. Previously, we have shown *Dientamoeba fragilis* and *Blastocystis* to be significantly correlated with healthy controls, thus disproving these as gastroenteritis pathogens [3,4].

Binary laboratory techniques such as (electron) microscopy, ELISA’s and stool cultures have been displaced by semi-quantitative molecular diagnostics. Molecular methods give the possibility to use Ct-values for differentiating clinically irrelevant positives from real positives [15,16]. Therefore, we performed a case-control study in our local patients visiting their GP to investigate the following:

- Establishing by PCR the prevalence of a broad range of presumptive-pathogenic viruses in stool samples from cases and controls.
- Whether semi-quantitative qRT-PCR results could discriminate real-positive from false-positive results in the diagnosis of gastroenteritis in the local GP population.
- Finally, to design an evidence-based multiplex molecular virus testing panel, tailored for our GP patients, and to compare it with available commercial multiplex panels.

## Materials and methods

### Study population

The study population was described previously [3] as; patients who visited the GP for GI complaints and for whom microbiologic examination was requested (cases), and a matched group of persons without GI complaints (controls). Matching criteria were age group (<5, 5–20, 21–50 and >50 years of age), month of sample collection, sex and region. GI complaints were defined as diarrhea and/or other abdominal discomfort for which an infectious cause is likely, as assessed by the GP.

Control subjects were either recruited by the GP (54%; consisting of patients visiting their GP for a variety of non-GI medical problems, all fitting criteria for an immunocompetent patient) or were healthy volunteers recruited by the laboratory (46%). Control subjects were excluded if they had experienced GI complaints within 4 weeks before sample collection.

In total, 2802 stool samples of case and control subjects were collected from August 2010 through December 2012.

### Ethics statement

Written approval was obtained by the medical ethics review board (Isala clinics, Zwolle, the Netherlands), and data for all samples were encoded to ensure anonymity according to the board’s requirements. Case and control subjects were requested to participate in the study by filling out a questionnaire and providing a fresh stool sample. All participants provided written informed consent. Examples of the questionnaires can be found in S1 File.

### Nucleic acid extractions

Total nucleic acids were extracted as described previously [3]: approximately 100μg frozen stool was suspended in 400μL STAR buffer (Roche), vigorously shaken on a Magnalyser (1 minute; Roche) and pelleted (3 minutes, 13 000 rpm). One-hundred microlitre of supernatant was extracted on the MagnaPure96 (MP96; Roche) using the DNA and Viral NA small volume kit, and total nucleic acids were eluted in 100 μL, phocine herpesvirus (PhHV) and equine arteritis virus (EAV) served as internal control. Eluates were stored at -80°C till tested.

### Polymerase chain reaction

An internally controlled multiplexed real-time PCR for viral pathogens was performed for adenovirus, astrovirus, bocavirus, enterovirus, norovirus GI and GII, parechovirus, rotavirus, salivirus, sapovirus, and torovirus. Primers and probes are listed in S1 Table. The results and PCRs for bacterial and protozoan microorganisms have been described previously [3].

Each assay was extensively validated regarding sensitivity, specificity, reproducibility and stability using analytical panels and clinical materials as well as international proficiency panels, if available. Torovirus was tested with virus cultures of Berne virus (equine torovirus), kindly provided by Erik J. Snijder (Leiden University Medical Center, The Netherlands). No clinical salivirus samples were available, hence primer and probe sequences were BLASTed against known genomic sequences, and were validated using a synthetic DNA construct spanning position 6839–6939 of the salivirus genome (MK801667.1).

For each reaction, 7.5μL 4× Fast Virus mastermix (Life Technologies, USA) was mixed with Bovine serum albumin (12μg/reaction; Life Technologies, USA), dUTP (6nmole/reaction, Roche) and Uracil N-glycosylase (0.3U/reaction, Roche). Oligos diluted in 1xTE (Sigma-Aldrich) and 10μL of DNA/RNA extract were added to the master mix to form a total reaction volume of 30μL.

Most detections were executed with the CFX96 real-time PCR Detection System (Bio-Rad) with the following program: 50°C for 5 minutes, 95°C for 20 seconds, followed by 45 cycles of 95°C for 15 seconds, 57°C for 30 seconds and 60°C for 30 seconds including plate read. Salivirus and torovirus were detected with the ABI7500 real-time thermocycler (Life Technologies), using the following program: 50°C for 5 minutes, 95°C for 20 seconds, followed by 45 cycles of 95°C for 15 seconds and 60°C for 60 seconds.

### Adenovirus typing

Adenovirus typing was performed as described previously [17] by sequencing the variable region 7 of the hexon gene, using an ABI 3500 Genetic analyzer (Life Technologies) and BigDye Terminator cycle sequencing kit (Applied Biosystems). Adenovirus types were determined by comparing sequences to reference adenovirus sequences using Clustal X2.1 and seaview 4.3.3 [18,19].

### Data analysis

Continuous data were presented as mean with standard deviation (SD), categorical variables as number with corresponding percentage. Differences between the cases and controls were analyzed using independent samples t-test for continuous data and Chi-square test for categorical data. Univariate and multivariate logistic regression analyses were performed to calculate odds ratios (OR) with 95% confidence intervals (CI). Adjusted OR (aOR) were calculated after the database was adjusted for differences in age, sex, and recent travel abroad. A p-value <0.05 was regarded as being statistically significant. Statistical analyses were performed using the Statistical Package for the Social Sciences version 24 (SPSS Inc., Chicago, USA).

## Results

**Fig 1:**
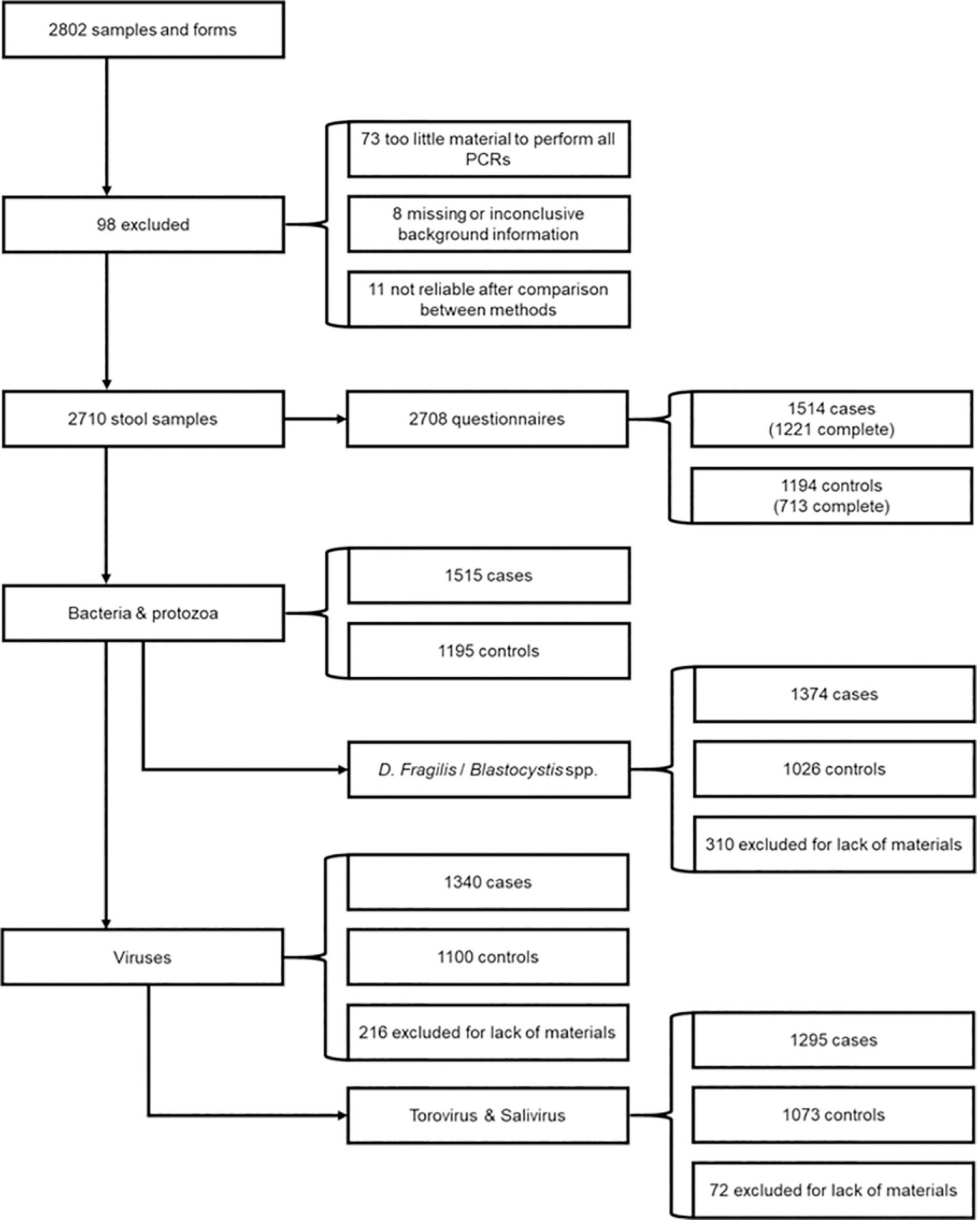
Flow scheme of the materials used in this study and [3,4].

In the original study [3], 2710 samples were included: 1515 cases and 1195 controls. Since some samples were used up in the previous studies [3,4] not all samples were available for subsequent analysis of the classic viruses (i.e. adenovirus group F (adenovirus-F), norovirus GI and GII, rotavirus, sapovirus) (Fig 1, Table 1, N = 216 excluded), or torovirus and salivirus (Fig 1, N = 288 excluded).

**Table 1:** Prevalence of the investigated viruses in the general population with symptoms of gastroenteritis (cases, N = 1340) and those without symptoms (controls, N = 1100).

	cases (n = 1340)	controls (n = 1100)	OR (95% CI) unadjusted	p-value	aOR (95% CI) *	p-value
Adenovirus-non-F	57 (4.3%)	34 (3.1%)	1.39 (0.90–2.15)	0.133	1.20 (0.75–1.91)	0.444
*Subtype*						
C1	9 (0.7%)	3 (0.3%)	2.47 (0.67–9.16)	0.175		
C2	13 (1.0%)	5 (0.5%)	2.15 (0.76–6.04)	0.148		
C5	2 (0.1%)	1 (0.1%)	1.64 (0.15–18.14)	0.685		
A31	2 (0.1%)	3 (0.3%)	0.55 (0.09–3.28)	0.509		
B3	1 (0.1%)	0 (0%)	-	-		
D17	1 (0.1%)	0 (0%)	-	-		
D43	1 (0.1%)	0 (0%)	-	-		
Unknown	28 (2.1%)	22 (2.0%)	1.05 (0.60–1.84)	0.877		
Adenovirus-F	9 (0.7%)	1 (0.1%)	7.43 (0.94–58.75)	0.057	6.37 (0.80–50.92)	0.081
Astrovirus	13 (1.0%)	1 (0.1%)	10.77 (1.41–82.43)	0.022	10.37 (1.34–80.06)	0.025
Bocavirus	1 (0.1%)	1 (0.1%)	0.82 (0.05–13.14)	0.889	0.85 (0.05–13.64)	0.905
Enterovirus	42 (3.1%)	33 (3.0%)	1.05 (0.66–1.66)	0.848	0.83 (0.50–1.37)	0.462
Noroviruses	61 (4.6%)	17 (1.5%)	3.04 (1.76–5.23)	<0.001	2.88 (1.66–5.01)	<0.001
Nov GI	15 (1.1%)	5 (0.5%)	2.48 (0.90–6.84)	0.080	2.22 (0.79–6.23)	0.128
Nov GII	46 (3.4%)	12 (1.1%)	3.22 (1.70–6.12)	<0.001	3.10 (1.62–5.92)	0.001
Parechovirus	10 (0.7%)	5 (0.5%)	1.65 (0.56–4.83)	0.364	1.61 (0.54–4.77)	0.395
Rotavirus	10 (0.7%)	0 (0.0%)	-	-	-	-
Sapovirus	35 (2.6%)	23 (2.1%)	1.26 (0.74–2.14)	0.401	1.15 (0.67–1.98)	0.606

Cases are defined as persons having symptoms of gastroenteritis or persons where the general practitioner suspected gastroenteritis due to infectious cause (N = 1340) and controls are defined as people without symptoms (N = 1100). aOR: adjusted OR. Data are presented as n (%).

*Adjusted for age, sex, and recent travel abroad.

The population studied in this report was not significantly different from the original population (Fig 1, S2 Table); females constituted 56.7% and 56.5% of the cases and controls, respectively. Diarrhea was noted in 92.8% of cases and none in controls (S2 Table). Factors associated with gastrointestinal symptoms were antacid use (14.8% in cases *vs*. 8.6% in controls) and antibiotic use (6.6% in cases *vs*. 2.5% in controls). Moreover, cases more often reported recent travel abroad (14.9% in cases vs. 5.6% in controls) and household members with gastrointestinal symptoms (17.0% vs. 5.8%).

First, we tested for the presence of torovirus and salivirus in 2368 samples which had material available for the analysis (1295 cases, 1073 controls): no torovirus was found, whereas we found 11 samples positive for salivirus: 8 cases (0.6%) and 3 controls (0.3%) (adjusted OR (aOR) 1.67, 95% confidence interval (CI): 0.43–6.54, p-value: 0.461).

Having excluded an association of salivirus or torovirus with gastroenteritis in the GP population, we analyzed the association between other viruses with gastroenteritis; adenovirus-F and other adenovirus-types (adenovirus-non-F), astrovirus, bocavirus, enterovirus, norovirus GI and GII, parechovirus, rotavirus and sapovirus (Table 1).

The prevalence of any virus in cases and controls was 14.9% (199/1340) and 7.2% (79/1100), respectively (Table 1). Considering only the youngest age-category of <5 years, positivity for any virus was 55.2% in cases (74/134) and 45.8% in controls (44/96). The most prevalent viruses were adenoviruses (5.0% in cases and 3.2% in controls (N = 66 and 35, respectively)) and noroviruses (4.6% in cases and 1.5% in controls (N = 61 and 17, respectively)) See also S1 Fig.

Co-infections with multiple viruses were rare (prevalence of 2.6% in cases (35/1338) and 1.4% in controls (15/1092), respectively). In contrast: co-infections of a virus with a bacteria or parasite were more common (82/1338; 6.1%) in cases than in controls (35/1092; 3.2%). In our first studies, we found 35.4% of the cases (473/1338) and 18.3% of the controls (200/1092) were positive for any of the studied bacteria or parasites [3].

Moreover, the aOR in Table 1 shows that ‘classic’ gastroenteritis viruses had higher prevalence in cases than in controls; Adenovirus-F (aOR 6.37, 95% CI: 0.80–50.92, p = 0.081), astrovirus (aOR 10.37, 95% CI: 1.34–80.06, p = 0.025), norovirus GI (aOR 2.22, 95% CI: 0.79–6.23, p = 0.128), norovirus GII (aOR 3.10, 95% CI: 1.62–5.92, p = 0.001) (Table 1). Bocavirus, enterovirus, parechovirus and sapovirus were not statistically significantly associated with gastroenteritis cases in our population (p>0.05).

Adenoviruses were detected by two PCRs; one for adenovirus group F and the other for adenovirus-broad detection. Adenovirus-F are typically associated with gastroenteritis (Table 1). In case of adenovirus-non-F, the prevalence was higher in cases than in controls, however not significant. The association of adenovirus with gastroenteritis or other ailments can be type-specific [20,21]. To elucidate whether specific adenovirus types are relevant in the context of gastroenteritis, adenovirus-positive samples were typed by sequencing. Many adenovirus-positive samples were non-typeable (28/57 and 22/34 in cases and controls, respectively), due to a low load, i.e. high Ct value. Adenovirus-C (C1, C2, C5) was the most prevalent type (65%; 33/51).

**Fig 2:**
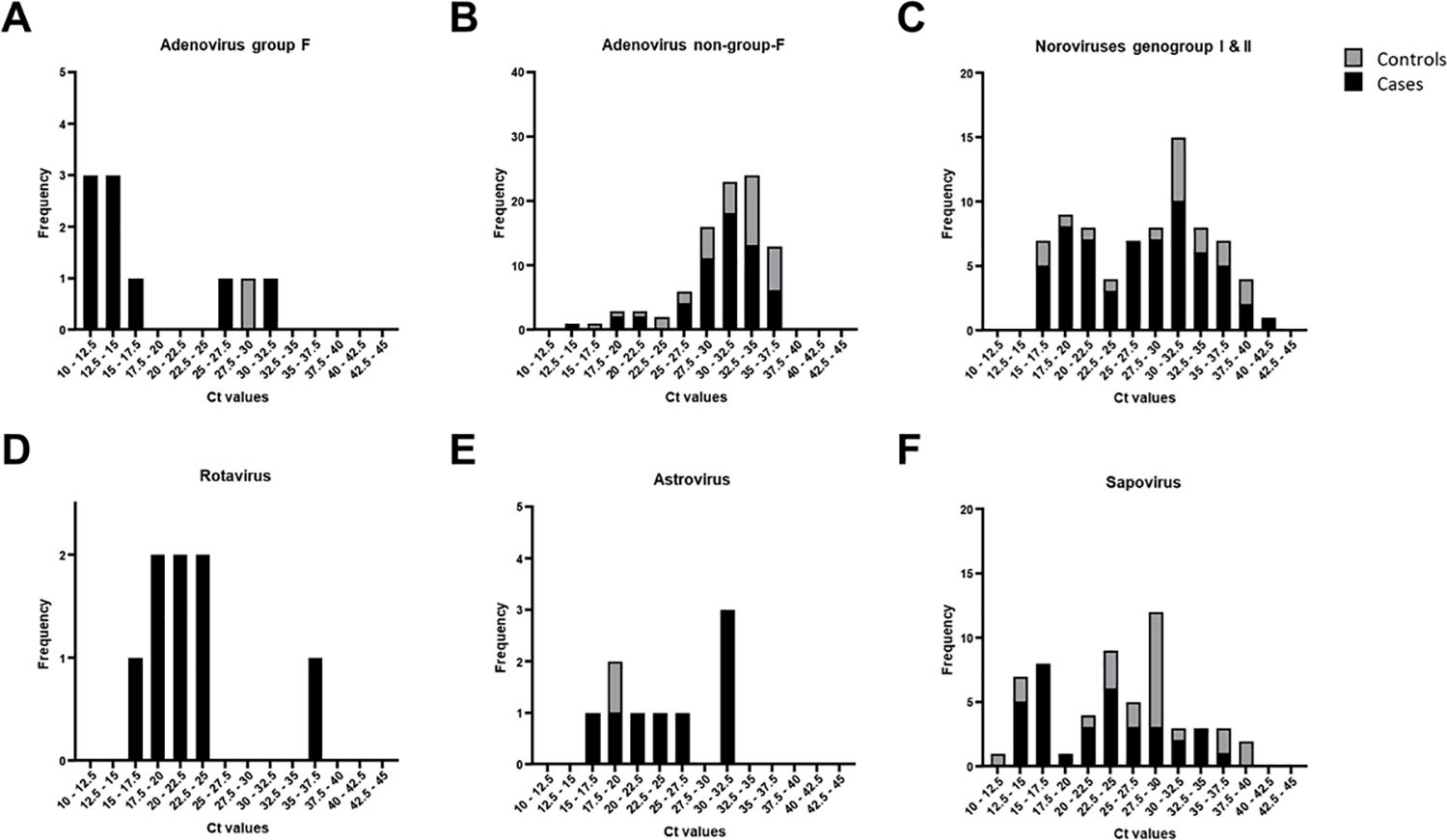
Distribution and frequency of PCR positive samples for astrovirus, Adenovirus non-group-F, adenovirus group F, sapovirus, rotavirus, and noroviruses (genogroup I & genogroup II). Shown are the results for adenovirus-F (A), adenovirus-non-F (B), noroviruses (C), rotavirus (D), astroviru (E) and sapovirus (F). Shown is the number of positive materials from cases (black bars) and controls (grey bars) per category of Ct values (bin size 2.5 Ct).

The viral load has been proposed as a factor in the pathogenesis of several viruses, including norovirus [22,23]. Therefore, we investigated whether the semi-quantitative qRT-PCR could discriminate between carriage (i.e. controls) and disease (i.e. cases) (Fig 2A–2F). However, for none of the investigated viruses this appeared to be the case.

Previously, we detected several bacterial and protozoal pathogens [3]. To see whether the significance of the viral pathogens depended on the presence of other (proven) pathogens, we reanalyzed our database after omitting all subjects with a proven pathogen (S3 Table). The significance of astrovirus, norovirus GII and rotavirus was maintained. The p value for adenovirus-F and norovirus GI remained above 0.05, however this was most likely because of low numbers of positive subjects rather than the co-presence of these viruses with other proven pathogens.

## Discussion

In The Netherlands, approximately 4.5 million people a year experience an episode of gastroenteritis [1], and approximately 5% thereof visit their GP for gastrointestinal complaints [2]. Because the pathogen cannot be deduced from symptoms alone, laboratory diagnostics are essential. In particular, the field of molecular diagnostics has made significant impact in the timely diagnosis of GI complaints. Moreover, the introduction and application of metagenomics led to case reports with novel microorganisms [6]. However, case-control studies attributing these microorganisms to gastroenteritis in the general population were lacking. Therefore, we conducted a case-control study involving bacterial, protozoal and viral pathogens. Following our analyses on the bacterial and protozoal agents we now report on the viruses [3,4] and focused on a broad range of 11 viruses with a literature-proven or suspected gastrointestinal pathogenesis. We found that, in our population of GP patients with gastroenteritis, adenovirus-F, astrovirus, norovirus GI and GII, and rotavirus were related to cases.

To our knowledge, only two case-control studies have been conducted in the general population of a HIC [24,25]. They employed older methods, e.g. electron microscopy or ELISA [5,26], or were focusing on different patient populations [27,28] or developing countries [29], in contrast to ours, which used the most sensitive and specific method available for all detections (i.e. qRT-PCR) [29]. Our study employed a more sensitive and specific method (qRT-PCR) and included novel viruses as e.g. salivirus, torovirus and bocavirus.

The prevalence of any virus in cases and controls was 14.8% and 7.2%, respectively. Overall, the most prevalent viruses were adenovirus and noroviruses. Strikingly, in the age category <5 years, half of the children were positive for any virus, in line with previous reports [22,30], indicating that (asymptomatic) carriage of viruses is common in this age group. The significant higher prevalence in cases vs. controls for norovirus GI and GII, rotavirus, astrovirus and adenovirus-F (i.e. the ‘classic’ gastrointestinal viruses) are in agreement with the literature, which validates both the selected population and the methodology used in this study. Although a higher prevalence was observed in cases for adenovirus-non-F, parechovirus, sapovirus and enterovirus, it did not reach significancy. Torovirus, bocavirus and salivirus, not or only sparsely encountered in our study population, were not statistically significantly related with cases. Both astrovirus and rotavirus were related statistically significantly with cases, with high positive predictive values of 93% and 100%, respectively. Sapovirus, though more frequent, was not significantly related with cases, neither in the study population at large nor in children aged <5 years. Although solid case-control studies are lacking, sapovirus has been reported to be statistically significantly associated with gastroenteritis in the elderly and/or in long-term care facilities [28,31]. Since, these sub-populations are typically not served by general practitioners in the Netherlands, elderly or long-term care residents are not included in this study.

As in our study, both symptomatic as well as asymptomatic carriage has been described of norovirus GI and GII [22,30]. Even serum RNA load has been correlated significantly with increased fecal viral load in children [23]. However, the Ct value could not differentiate symptomatic cases from asymptomatic carriers of norovirus and sapovirus. Therefore, positive samples should be reported and subsequently interpreted in the context of the clinical context.

In the Dutch GP population, adenovirus-F is statistically significantly associated with symptomatic gastroenteritis, while adenovirus-non-F are not. Recently, adenovirus-C1, C2, C5 and C6 were shown to be associated with gastroenteritis in some positive subjects [32], in line with previous findings in HIC [32,33]. In this study, adenovirus-C1, C2 and C5 indeed have higher OR, yet lacked significancy due to the low number of positives. More research is needed to elucidate the gastroenteric potency of adenovirus-C types.

Our results are of practical use when deciding which viruses to include in a test panel for patients with gastroenteritis from our HIC GP population. Based on our findings, our panels should comprise adenovirus-F, astrovirus, norovirus GI and GII, and rotavirus. Our panel, based on the epidemiological evidence presented in this study, differs from several popular, commercial panels (Table 2 below) [34]. This is of relevance, as per May 26^th^, 2022, the EU regulation on *in vitro* diagnostic medical devices will come into effect, with the consequence that in-house molecular tests will likely be displaced by commercial alternatives. Based upon our study, we would replace the in-house panel with an alternative which tests for adenovirus-F, astrovirus, norovirus GI and GII, and rotavirus. Inclusion of sapovirus is not necessary for our HIC GP population with gastrointestinal complaints.

**Table 2:** Composition of viral pathogens of Isala and, respectively, commercial multiplex gastroenteritis panels.

	Multiplex panel				
Viruses	Isala	FilmArray GI	xTAG® GPP	Verigene EP	BD MAX™ EV
Adenovirus F40 / 41	✓	✓	✓		✓
Astrovirus	✓	✓			✓
Norovirus GI / GII	✓	✓	✓	✓	✓
Rotavirus A	✓	✓	✓	✓	✓
Sapovirus (I, II, IV en V)		✓			✓

Commercial panels: FilmArray GI (BioMérieux Benelux BV, Amersfoort, The Netherlands); xTAG® GPP and Verigene EP (Luminex, ’s-Hertogenbosch, The Netherlands); BD MAX™ EV (Becton-Dickinson, Vianen, The Netherlands).

More importantly, our results argue against blind testing of patients against all presumptive enteropathogenic viruses. In fact, broad screening may produce clinically false-positive findings as seen for enterovirus and parechovirus. In children <5 years, the combined prevalence of enterovirus and parechovirus was almost 25% in cases as well as controls. This should be considered when screening fecal samples from young children. Case-reports had pointed at potential enteropathogenicity of viruses like bocavirus, salivirus and torovirus. We show that these viruses have a low prevalence in our GP population and should not be included in the first-line screening.

In conclusion, we found that, in our population of GP patients with gastroenteritis, adenovirus-F, astrovirus, norovirus GI and GII, and rotavirus were significantly related to cases, in contrast to adenovirus-non-F, parechovirus, sapovirus and enterovirus. Torovirus, bocavirus and salivirus were not or only sparsely encountered, and are not related with cases. Ct values of the PCRs did not differentiate between cases and controls. Finally, the commercial panels, currently out there in the marketplace, are not the best suited choice for testing our GP population, either because they lack astrovirus or include sapovirus.

## Supporting information

S1 Fig Prevalence of viruses in children <5 years versus all other participants. (DOCX)

S1 Table Primers and probes used in this study. (DOCX)

S2 Table Characteristics of the cases and controls, and data retrieved from questionnaires. (DOCX)

S3 Table Prevalence of viruses in absence of other, proven pathogenic microorganisms. (DOCX)

S4 Table Anonymized database used in the Case Control study for gastroenteritis in the general population of the Netherlands (2010–2012). (XLSX)

S1 File Examples of the questionnaires for controls and for cases. (DOCX)
