# Supplementary material for: From a case-control survey to a diagnostic viral gastroenteritis panel for testing of general practitioners’ patients
Source: PLoS One. 2021 Nov 3;16(11):e0258680. doi: 10.1371/journal.pone.0258680 (PMC8565752; doi:10.1371/journal.pone.0258680)
Supplement: S1 Fig — (DOCX) [file pone.0258680.s001.docx]

**
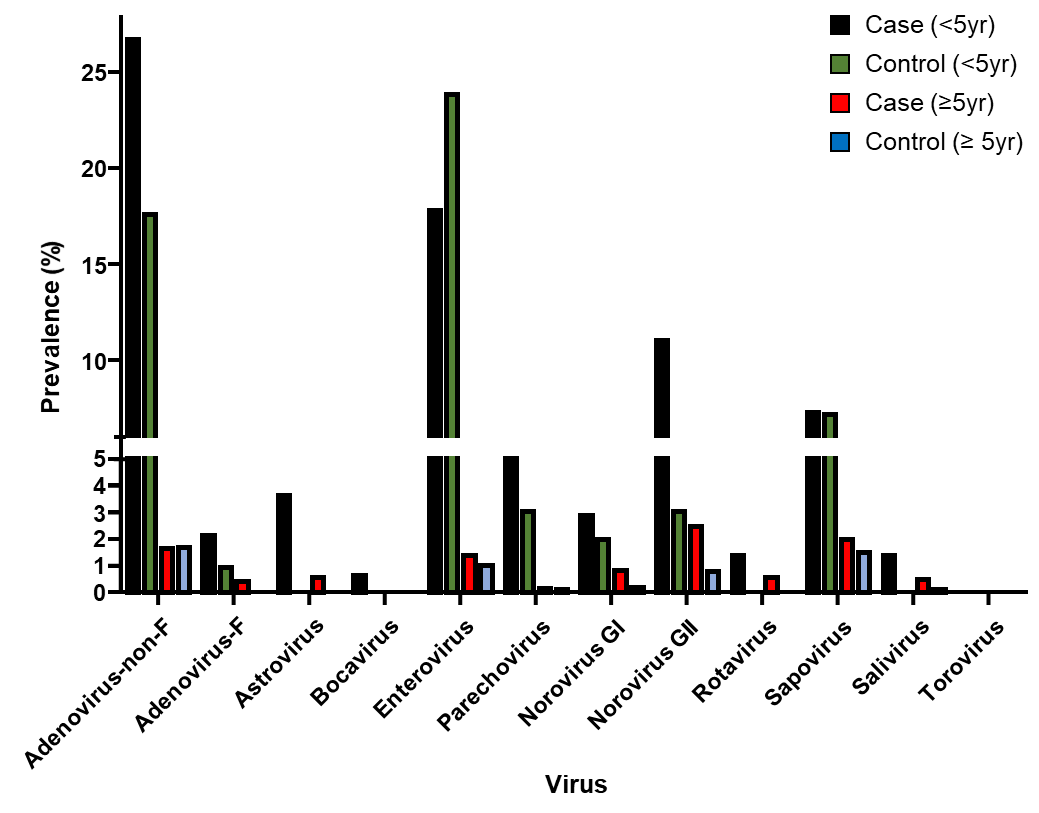
**

**S1 Figure. Prevalence of viruses in children younger than 5 years versus older participants.**

The prevalence of viruses is shown for participants younger than 5 years (<5yr) and in all other participants (≥5yr), each divided in cases and controls. N= 135, 96, 1205, and 1004 respectively. Shown is the prevalence of adenovirus non-group F, adenovirus group F, astrovirus, bocavirus, enterovirus, parechovirus, norovirus GI, norovirus GII, rotavirus, sapovirus, salivirus and torovirus.
