# Supplementary material for: From a case-control survey to a diagnostic viral gastroenteritis panel for testing of general practitioners’ patients
Source: PLoS One. 2021 Nov 3;16(11):e0258680. doi: 10.1371/journal.pone.0258680 (PMC8565752; doi:10.1371/journal.pone.0258680)
Supplement: S1 Table — (DOCX) [file pone.0258680.s002.docx]

**S1 Table. Primers and probes used in this study.**

Target organism Gene pmol/reaction Name of oligo Sequence Reference multiplex

Adenovirus Hexon 10 Fadv1 CATGACTTTTGAGGTGGATC [1] 1

10 Fadv31 TATGACATTTGAAGTTGACC

10 Fadv4 CATGAATTTCGAAGTCGACC

10 Radv CCGGCCGAGAAGGGTGTGCGCAGGTA

6 Padv AGCCCACCCTKCTTTAT-MGB-FAM

6 Padv4 GAGTCYACCCTTCTCTATGT-MGB-FAM

Adenovirus F40/41 Hexon 10 FadvF CTCGACATGACTTTTGAGGT [2] 1

10 RadvF GTAGACGGCCTCGATGAC

4,5 PadvF AGCCCACACTTCT-MGB-VIC

Astrovirus Orf1a 10 Fasv TCTYATAGACCGYATTATTGG [2] 2

20 Rasv TCAAATTCTACATCATCACCAA

9 Pasv CCCCADCCATCATCATCTTCATCA-TQ-VIC

Bocavirus NP-1 10 Fbov GGAAGAGACACTGGCAGACAA [3] 1

10 Rbov GGGTGTTCCTGATGATATGAGC

4.5 Pbov CTGCGGCTCCTGCTCCTGTGAT-TQQua705

Enterovirus 5’-UTR 20 Fenv2 GACATGGTGYGAAGAGTCTATTGA [4] 3

20 Renv GATTGTCACCATAAGCAGCCA

9 Penv2 FAM CGGAACCGACTACTTTGGGTGTCCGTGTTTC BHQ1

Norovirus GI ORF1/ORF2 27 FnovI CGYTGGATGCGNTTYCATGA [2] 4

27 RnovI CCTTAGACGCCATCATCATTTAC

10,2 PnovI-MGB-VIC TYGCGRTCTCCTGTCCA-MGB-VIC

Norovirus GII ORF1/ORF2 10 FnovII CARGARBCNATGTTYAGRTGGATGAG [2] 2

20 RnovII TCGACGCCATCTTCATTCACA

10,5 PnovII AGATYGCGATCSCCCTC-MGB-FAM

Parechovirus 5’-UTR 20 Fpev AAACACTAGTTGTAAGGCCCA *personal communication* 3

20 Rpev1 GTTTGGCCCACTAGACGTT

7,5 Ppev VIC GAAGGATGCCCAGAAGGTACCCG BHQ1

Rotavirus NSP3 10 Frov ACCATCTTCACGTAACCCTC [2] 4

10 Frov2 ACCATCTACACATGACCCTC

10 Rrov CACATAACGCCCCTATAGCC

4,5 Prov ATGAGCACAATAGTTAAAAGCTAACACTGTCAA-TQ-Qua705

Salivirus 5’UTR 10 Fsali2 TCTGCTTGGTGCCAACCTC [5] 5

10 Rsali2 CCARGCACACACATGAGRGGATAC

4,5 Psali2 TGCGGGAGTGCTCT-MGB-FAM

Sapovirus orf1 10 Fsav124 GAYCASGCTCTCGCYACCTAC [6] 4

10 Fsav1 TTGGCCCTCGCCACCTAC

10 Fsav5 TTTGAACAAGCTGTGGCATGCTAC

10 Rsav1245 CCCTCCATYTCAAACACTA

4,5 Psav124 CCRCCTATRAACCA-MGB-FAM

4,5 Psav5 TGCCACCAATGTACCA-MGB-FAM

Torovirus N 10 Ftoro TAATGGCACTGAAGACTCTAATC [7] 5

10 Rtoro ACATAACATCTTACATGGAGACAC

4,5 Ptoro GCCTCTGGCTCCACTGTGTG-TQ-VIC

Equine arteritis virus orf1a 10 Feav2 TCTCTTGCTTTGCTCCTTAGC this publication

10 Reav2 AACAACATTATTGCCCACACTG

4,5 Peav2 TTGCCATTGGGTTGATA-MGB-Cy5

Parechovirus – personal communication: Radboud UMC (Arjan de Jong) and Izore (Rianne van Ree).

**References**

1. Loens K, van Loon AM, Coenjaerts F, van Aarle Y, Goossens H, Wallace P, Claas EJ, Ieven M, GRACE Study Group. Performance of different mono- and multiplex nucleic acid amplification tests on a multipathogen external quality assessment panel. J Clin Microbiol 2012; 50: 977-987.

2. van Maarseveen NM, Wessels E, de Brouwer CS, Vossen AC, Claas EC. Diagnosis of viral gastroenteritis by simultaneous detection of Adenovirus group F, Astrovirus, Rotavirus group A, Norovirus genogroups I and II, and Sapovirus in two internally controlled multiplex real-time PCR assays. J Clin Virol 2010; 49: 205-210.

3. Allander T, Jartti T, Gupta S, Niesters HG, Lehtinen P, Osterback R, Vuorinen T, Waris M, Bjerkner A, Tiveljung-Lindell A, van den Hoogen, B G, Hyypia T, Ruuskanen O. Human bocavirus and acute wheezing in children. Clin Infect Dis 2007; 44: 904-910.

4. Hoek RA, Paats MS, Pas SD, Bakker M, Hoogsteden HC, Boucher CA, van der Eerden, M M. Incidence of viral respiratory pathogens causing exacerbations in adult cystic fibrosis patients. Scand J Infect Dis 2013; 45: 65-69.

5. Yu JM, Ao YY, Liu N, Li LL, Duan ZJ. Salivirus in Children and Its Association with Childhood Acute Gastroenteritis: A Paired Case-Control Study. PLoS One 2015; 10: e0130977.

6. Oka T, Katayama K, Hansman GS, Kageyama T, Ogawa S, Wu FT, White PA, Takeda N. Detection of human sapovirus by real-time reverse transcription-polymerase chain reaction. J Med Virol 2006; 78: 1347-1353.

7. Duckmanton L, Luan B, Devenish J, Tellier R, Petric M. Characterization of torovirus from human fecal specimens. Virology 1997; 239: 158-168.
