# Supplementary material for: From a case-control survey to a diagnostic viral gastroenteritis panel for testing of general practitioners’ patients
Source: PLoS One. 2021 Nov 3;16(11):e0258680. doi: 10.1371/journal.pone.0258680 (PMC8565752; doi:10.1371/journal.pone.0258680)
Supplement: S2 Table — (DOCX) [file pone.0258680.s003.docx]

**S2 Table. Characteristics of the cases and controls, and data retrieved from questionnaires**

|  | | Cases (n = 1340) | Controls (n = 1100) | P value |
| --- | --- | --- | --- | --- |
| Age category^a^ | | 1340 (100) | 1090 (100) | 0.075 |
|  | <5 years | 135 (10.1) | 96 (8.8) |  |
|  | 5-20 years | 269 (20.1) | 187 (17.2) |  |
|  | 21-50 years | 500 (37.3) | 406 (37.2) |  |
|  | >50 years | 436 (32.5) | 401 (36.8) |  |
| Female^b^ | | 760 (56.7) | 617 (56.5) | 0.895 |
| Recent travel abroad^c^ | | 200 (14.9) | 62 (5.6) | <0.001 |
| Household members with GI symptoms^d^ | | 191 (17.0) | 38 (5.8) | <0.001 |
| Antacid use^e^ | | 197 (14.8) | 95 (8.6) | <0.001 |
| Antibiotic use^f^ | | 87 (6.6) | 27 (2.5) | <0.001 |
| Disease related characteristics | |  |  |  |
| Diarrhea^g^ | | 1078 (100) | NA |  |
|  | No diarrhea | 78 (7.2) |  |  |
|  | Total diarrhea | 1000 (92.8) |  |  |
|  | <1 week diarrhea | 169 (15.7) |  |  |
|  | 1-2 weeks diarrhea | 265 (24.6) |  |  |
|  | >2 weeks diarrhea | 566 (52.5) |  |  |
| Abdominal pain/cramps^h^ | | 824 (69.0) |  |  |
| Fever^i^ | | 156 (13.1) |  |  |
| Vomiting^j^ | | 150 (12.6) |  |  |
| Blood in stool^k^ | | 95 (8.0) |  |  |
| Mucus in stool^l^ | | 261 (22.0) |  |  |
| Data are presented as n (%)  Number of questionnaires with missing answers study population: ^a^controls n=10; ^b^controls n=7; ^c^cases n=1; controls n=1; ^d^cases n=215; controls n=448; ^e^cases n=13; controls n=1; ^f^cases n=17; controls n=3; ^g^n=262; ^h^n=145; ^i^n=152; ^j^n=154; ^k^n=154; ^l^n=156. NA not applicable. GI Gastrointestinal | | | | |
