# Supplementary material for: From a case-control survey to a diagnostic viral gastroenteritis panel for testing of general practitioners’ patients
Source: PLoS One. 2021 Nov 3;16(11):e0258680. doi: 10.1371/journal.pone.0258680 (PMC8565752; doi:10.1371/journal.pone.0258680)
Supplement: S3 Table — (DOCX) [file pone.0258680.s004.docx]

**S3 Table. Prevalence of viruses in absence of other, established pathogenic microorganisms.**

|  | **Prevalence in cases** | **Prevalence in controls** | **p-value Chi square test** | **Number of samples positive for ≥1 other pathogen *** |
| --- | --- | --- | --- | --- |
| Adenovirus | 43 (3.3%) | 35 (3.2%) | 0.930 | 37 |
| *Subtype* |  |  |  |  |
| 1C | 5 (0.4%) | 1 (0.1%) | 0.232 | 2 |
| 2C | 3 (0.2%) | 2 (0.2%) | 1.000 | 4 |
| 31A | 1 (0.1%) | 2 (0.2%) | 0.592 | 1 |
| 43D | 0 | 0 |  | 1 |
| 5C | 1 (0.1%) | 1 (0.1%) | 1.000 | 0 |
| Unknown | 33 (2.5%) | 29 (2.7%) | 0.817 | 29 |
| Adenovirus F | 15 (1.1%) | 5 (0.5%) | 0.069 | 5 |
|  |  |  |  |  |
| Astrovirus | 11 (0.8%) | 0 (0.0%) | 0.001 | 3 |
| Bocavirus | 1 (0.1%) | 1 (0.1%) |  | 0 |
| Enterovirus | 22 (1.7%) | 21 (1.9%) | 0.625 | 33 |
| Norovirus GI | 11 (0.8%) | 5 (0.5%) | 0.260 | 7 |
| Norovirus GII | 33 (2.5%) | 13 (1.2%) | 0.019 | 17 |
| Parechovirus | 4 (0.3%) | 5 (0.5%) | 0.740 | 6 |
| Rotavirus | 8 (0.6%) | 0 (0.0%) | 0.010 | 2 |
| Salivirus | 5 (0.4%) | 2 (0.2%) | 0.467 | 4 |
| Sapovirus | 28 (2.1%) | 17 (1.6%) | 0.321 | 13 |
| Torovirus | - | - |  |  |

* Positive for the reported virus and at least one of the following, established pathogens[8]; *Giardia lamblia, Cryptosporidium parvum, Entamoeba histolytica*, Campylobacter species, *Salmonella enterica, Yersinia enterocolitica*, pathogenic *E. coli* (Entero-invasive, Shigella toxin positive, enterohaemorrhagic, enteroaggregative, typical and atypical enteropathogenic). *Clostridioides difficile* is considered pathogenic in those 5 years and older.
