# Supplementary material for: From a case-control survey to a diagnostic viral gastroenteritis panel for testing of general practitioners’ patients
Source: PLoS One. 2021 Nov 3;16(11):e0258680. doi: 10.1371/journal.pone.0258680 (PMC8565752; doi:10.1371/journal.pone.0258680)
Supplement: S1 File — (DOCX) [file pone.0258680.s006.docx]

Informed consent and questionnaire CCGE study – Control

(note: CCGE = Case Control study Gastro-Enteritis)

1. Do you know of any people with diarrhea in your vicinity?

No

Yes If Yes, who? (Child, partner, etc.)

……………………….

……………………….

1. Did you make any trips abroad in the last month?

No

Yes If Yes, which countries?

…………………………………………..

1. Do you use any of the following medications?

Antibiotics If yes:

Which? …... from …… till …….

Antacids If yes:

Which? ……… from …….. till ………

Questionnaire CCGE study – Case

(note: CCGE = Case Control study Gastro-Enteritis)

1. Which complaints do you have besides diarrhea? (more than one answer possible)

Fever

Abdominal pains

Vomiting

1. Since when do you have diarrhea? ……………….
2. What is the aspect of the feces? (more than one answer possible)

Mushy contains mucus

Watery bloody

1. Do you have any suspicions what caused your diarrhea?
2. Do you know of any people with diarrhea in your vicinity?

No

Yes If Yes, who? (Child, partner, etc.)

……………………….

……………………….

1. Did you make any trips abroad in the last month?

No

Yes If Yes, which countries?

…………………………………………..

1. Do you use any of the following medications?

Antibiotics If yes:

Which? …... from …… till …….

Antacids If yes:

Which? ……… from …….. till ………
